# Theoretical analysis and design of hydro-hammer with a jet actuator: An engineering application to improve the penetration rate of directional well drilling in hard rock formations

**DOI:** 10.1371/journal.pone.0196234

**Published:** 2018-05-16

**Authors:** Jiang-fu He, Yun-pei Liang, Li-jia Li, Yong-jiang Luo

**Affiliations:** 1 State Key Laboratory of Coal Mine Disaster Dynamics and Control, Chongqing University, Chongqing, China; 2 Post-doctoral Research Station of Mineral Engineering, Chongqing University, Chongqing, China; 3 College of Resources and Environmental Science, Chongqing University, Chongqing, China; China University of Mining and Technology, CHINA

## Abstract

Rapid horizontal directional well drilling in hard or fractured formations requires efficient drilling technology. The penetration rate of conventional hard rock drilling technology in horizontal directional well excavations is relatively low, resulting in multiple overgrinding of drill cuttings in bottom boreholes. Conventional drilling techniques with reamer or diamond drill bit face difficulties due to the long construction periods, low penetration rates, and high engineering costs in the directional well drilling of hard rock. To improve the impact energy and penetration rate of directional well drilling in hard formations, a new drilling system with a percussive and rotary drilling technology has been proposed, and a hydro-hammer with a jet actuator has also been theoretically designed on the basis of the impulse hydro-turbine pressure model. In addition, the performance parameters of the hydro-hammer with a jet actuator have been numerically and experimentally analyzed, and the influence of impact stroke and pumped flow rate on the motion velocity and impact energy of the hydro-hammer has been obtained. Moreover, the designed hydro-hammer with a jet actuator has been applied to hard rock drilling in a trenchless drilling program. The motion velocity of the hydro-hammer ranges from 1.2 m/s to 3.19 m/s with diverse flow rates and impact strokes, and the motion frequency ranges from 10 Hz to 22 Hz. Moreover, the maximum impact energy of the hydro-hammer is 407 J, and the pumped flow rate is 2.3 m^3^/min. Thus, the average penetration rate of the optimized hydro-hammer improves by over 30% compared to conventional directional drilling in hard rock formations.

## 1. Introduction

In recent years, construction activities in hard rocks have extensively emerged with the practical increase of horizontal directional wells drilling technologies [1]. However, the penetration efficiency of the conventional hard rock drilling technology in horizontal directional well excavations is relatively low due to the multiple overgrinding of drill cuttings in bottom boreholes [2]. Existing drilling systems are only capable of drilling shallow and slim directional wells, which dramatically reduces the diameter of boreholes and indirectly increases the drilling cost [3]. Meanwhile, long drilling cycles, low penetration rates, and high drilling costs have impeded the development of the directional drilling technology for drilling in fractured, interbedded, and gravel-bearing rock formations [4, 5]. A new kind of drilling method with abrasive water jet and mechanical bit was developed by Tang because the dense “boss” in the hole bottom of hard rocks requires a more efficient drilling technique, and the new drilling method dramatically increases the drilling depth and efficiency compared to those of conventional drilling methods [6]. In addition, a hydraulic pulsed jet generator has been designed and field tested to reduce the abrasion of drill bit while drilling in medium to hard formations [7]. However, these kinds of drilling tools need more anti-abrasive nozzles, and their service life is unsatisfactory.

Given the low penetration efficiency of directional drilling in hard rocks, a specialized reamer has been applied to conventional trenchless technology [8]. Several attempts have been made to assess the drilling performance by correlating different rock properties with drilling rates [9, 10]. To improve the drilling efficiency of hard rocks, the physical mechanisms of rock fragmentation have also been studied by both theoretical and experimental methods [11, 12]. Greater penetration depth is obtained when more energy is inflicted on hard rocks.

The ratio of the crushing work to the impact energy per impact cycle has been numerically and experimentally investigated in crushing hard rocks with the percussive drilling technology, besides the energy conversion, transfer, and efficiency have also been analyzed [13–15], but the impact hammer energy has not been improved according to previous studies. In addition, the correlations between the penetration rate and properties of drilled hard rocks have been discussed [16, 17]. However, the influence of impact energy on penetration rate has not been considered. Moreover, the penetration rate of hard rocks drilling is influenced by geological, machine, and operating parameters [18]. Similarly, the influence of the impact energy of drilling tools on penetration rate are primarily investigated, especially in the horizontal directional well drilling in hard rock formations.

Therefore, new types of horizontal directional drilling tools should be innovatively proposed to improve the penetration rate in hard rock drilling, which could dramatically reduce the construction cost in the directional drilling of hard rocks. As one of the typical percussive and rotary drilling tools, hydraulic hammer has diverse advantages and extensive feasibility on horizontal directional drilling in complicated formations [19–21]. Due to the small diameter of boreholes and the low hydraulic energy efficiency and impact energy of hydro-hammers, most hydro-hammers have only been successfully used in shallow boreholes although percussion drilling technology is considered one of the best approaches for hard rock drilling. However, according to the literature, the most productive method of hard rock destruction is percussion-rotary technology [22]. By combining the rotary and percussive drilling technologies, a new hydro-hammer design with a jet actuator has been proposed and applied to horizontal directional drilling in hard rock formations.

To improve the impact energy of the hydro-hammer with a jet actuator, the operation methodology of the jet actuator has been demonstrated, and the motion velocity and impact energy of the hammer have been experimentally observed. Field tests using a hydro-hammer have been conducted in horizontal directional well drilling and showed the feasibility of using a hydro-hammer for drilling in hard rock formations.

## 2. Methodology of hydro-hammer with a jet actuator

**Fig 1:**
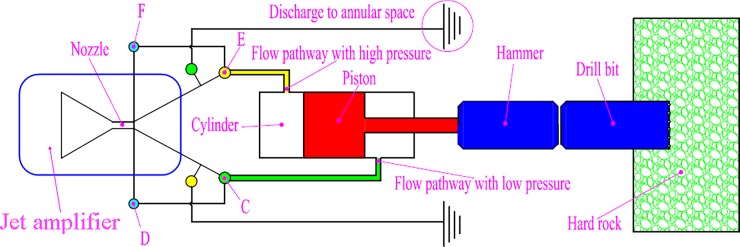
Schematic diagram of the hydro-hammer with a jet actuator in directional well drilling. C, E: Discharge channel; D, F: Controlled flow channel.

A hydro-hammer with a jet actuator is actuated by pressurized fluids (drilling mud or clean water), and the flushing nozzle of the jet actuator is the control component of the working performance of the hydro-hammer. As shown in Fig 1, while drilling a horizontal directional well in hard or complicated formations, pressurized fluids are pumped through the jet actuator, which is attached to one side of the actuator to access the cylinder. The pumped mud is attached to the upper chamber (green pipe line). Thus, the piston is actuated by high pressure mud to horizontally impact the drill bit. The controlled channel (F) is stimulated by a pressure pulse and the piston with the hammer moves to the right end-point, while the discharge channel of the high-pressure mud is shifted from channel E to channel C, and, thus, the mud is pumped into the lower chamber, which actuates the piston to move back to return strokes. The entire working process of the hydro-hammer rapidly and periodically completes in hundreds of microseconds. Therefore, the reciprocating impact energy transmission can be obtained by the periodic exchange of pumped mud between the upper and lower chambers.

Thus, the motion of the piston and hammer is determined by the variation of the pressure difference between the front and back chambers of the cylinder. Theoretically, the impact energy of the hydro-hammer can be obtained by taking the integral of impact forces and motion distance in addition to the impact forces that equal the product value of the pressure and action area. To improve the impact energy of the hydro-hammer with a jet actuator, the variation mechanism of the pressure difference between the front and back chambers of the cylinder must be theoretically analyzed.

**Fig 2:**
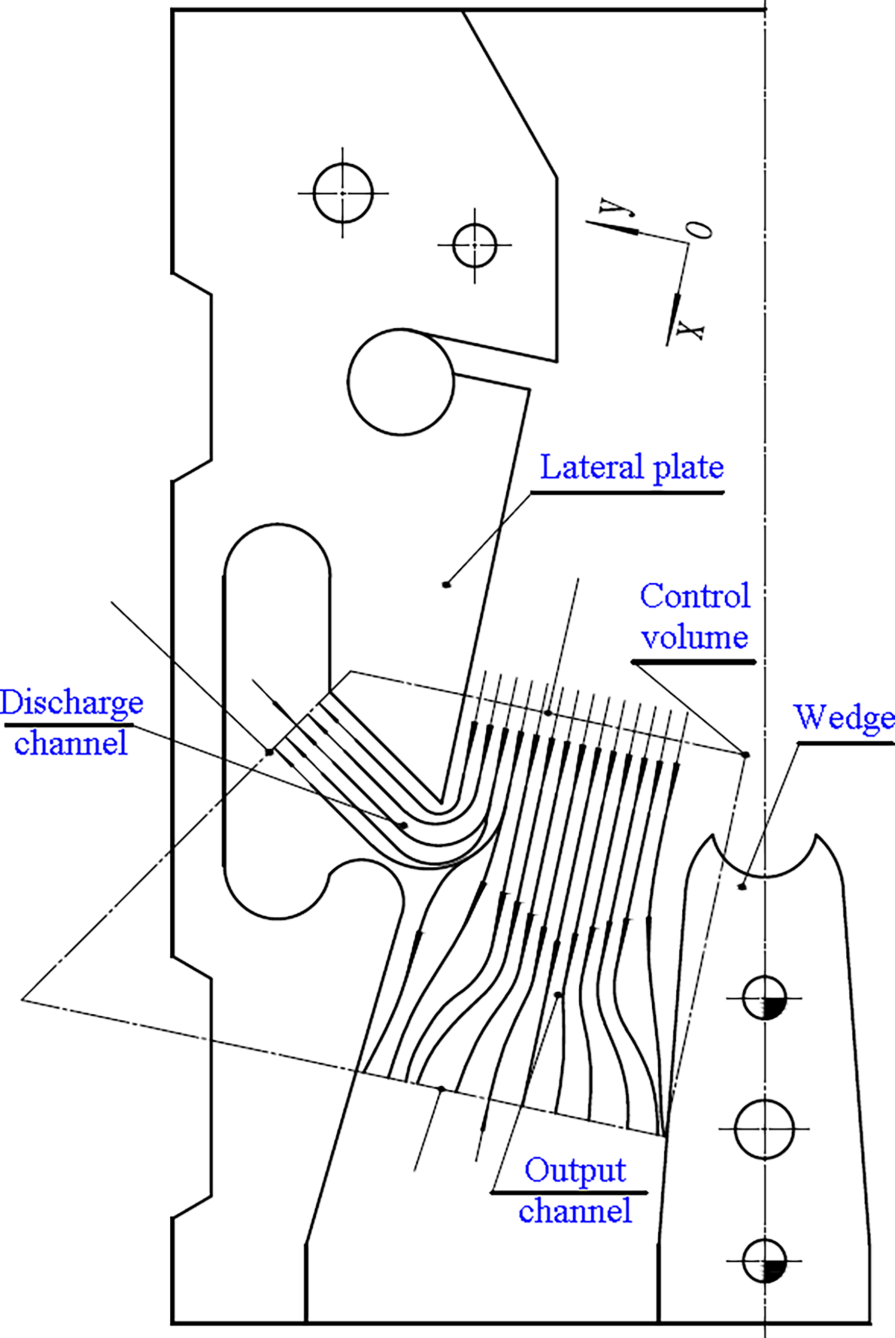
Schematic diagram of the fluid kinematics of a jet actuator in horizontal directional well drilling with hydro-hammers.

To establish the theoretical pressure model of the cylinder while in horizontal directional well drilling, the impulse hydro-turbine model has been utilized in the structure design of the hydro-hammer with a jet actuator. The control volume of the fluid in a jet actuator has been built, which consists of the axial jet from the nozzle, and the fluids flow through the discharge and output channels. As shown in Fig 2, the jet velocity and flow area of the fluids that flow through the nozzle are *V*_*j*_ and *S*_*j*_, respectively. Similarly, the velocity, flow area, and pressure of the fluids that flow through the discharge channel are denoted as *V*_*d*_, *S*_*d*_, and *P*_*d*_, respectively. In addition, the velocity, flow area, and pressure of the fluids that flow through the output channel are similarly denoted as *V*_*o*_, *S*_*o*_, and *P*_*o*_, respectively. According to the coordinate system, *xoy*, referring to the control volume, the instantaneous flow of the moving control volume is a constant value. To simplify the theoretical model, the mass force, static pressure, and friction of the control volume are ignored, and the comprehensive force that acts on the lateral plate of the jet actuator is set to zero. Therefore, the external forces that act on the control volume mainly include the hydraulic resistance (*R*_*d*_) from the discharge channel of the jet actuator and the hydraulic resistance (*R*_*o*_) from the output channel of the jet actuator. According to the continuity equation,

$$\int_{os}\rho {\bar{V}}_{r}•n•ds=0.$$ (1)

The loaded stress on the control volume can be obtained as follows:

$$V_{j}S_{j}=V_{d}S_{d}+V_{o}S_{o}.$$ (2)

On the basis of the momentum equation of incompressible fluid,

$$F=\int_{os}\rho V_{1r}•n•ds.$$ (3)

The force balance of the moving control volume in the *x* direction can be derived as follows:

$$-R_{o}+R_{d}\cos\theta =U_{1r}(-\rho V_{1r}S_{1})+U_{2r}(-\rho V_{2r}S_{2})$$ (4)

where

*R*_*o*_—Hydraulic resistance from the output channel of the jet actuator, opposite to the normal *x* direction;
*R*_*d*_—Hydraulic resistance from the discharge channel of the jet actuator;
*R*_*d*_ cos*θ*—Component of *R*_*d*_ in the *x* direction, *θ* is the angle between the discharge channel and the lateral plate of the jet actuator;
*U*_1*r*_—Velocity of the jet flow through the nozzle, *U*_1*r*_ = *V*_1*r*_;
*V*_1*r*_—Velocity of the moving control volume, *V*_1*r*_ = *V*_*j*_−*V*_*o*_;
*U*_2*r*_—Velocity of the fluids that flow through the discharge channel, *U*_2*r*_ *=* −*V*_2*r*_ cos*θ*;
*V*_2*r*_—Relative velocity of the jet flow through the discharge channel, which can be calculated by the velocity vector of the triangle, $\vec{V_{2r}}=\vec{V_{d}}-\vec{V_{o}}$;
*S*_1_, *S*_2_—Flow area of the jet flow through the nozzle and the discharge channel, *S*_1_ = *S*_*j*_, *S*_2_ = *S*_*o*_.

In addition, the relative velocity of jet flow through the discharge channel can be obtained by the cosine law.

$$V_{2r}=\sqrt{V_{d}^{2}+V_{o}^{2}+2V_{d}•V_{o}•\cos\theta }.$$ (5)

Therefore, Formula (4) can be transformed into Formula (6).

$$\begin{array}{l} R_{o}=R_{d}\cos\theta +(V_{j}-V_{o})\times [-\rho (V_{j}-V_{o})S_{j}]\\ \;+(-\sqrt{V_{d}^{2}+V_{o}^{2}+2V_{d}V_{o}\cos\theta }\times \cos\theta )\times \rho \sqrt{V_{d}^{2}+V_{o}^{2}+2V_{d}V_{o}\cos\theta }\times S_{d} \end{array}.$$ (6)

The hydraulic resistance (*R*_*d*_) from the discharge channel of the jet actuator can be derived by Formula (7).

$$R_{d}=P_{d}S_{d}=(\frac{1}{2}\xi_{9}\rho V_{d}{}^{2}+\frac{1}{2}\xi_{10}\rho V_{10}{}^{2}+\frac{1}{2}\xi_{11}\frac{L_{11}}{D_{h11}}V_{11}{}^{2}+P_{b})S_{d}.$$ (7)

The energy loss coefficient (*ξ*) can be acquired using the methods in the literature [23]. Thus, the hydraulic pressure of fluids that flow through the output channel can be presented as Formula (8).

$$\begin{array}{l} P_{o}=[(\frac{1}{2}\xi_{9}\rho V_{d}{}^{2}+\frac{1}{2}\xi_{10}\rho V_{10}{}^{2}+\frac{1}{2}\xi_{11}\frac{L_{11}}{D_{h11}}V_{11}{}^{2}+P_{b})S_{d}\cos\theta \\ \;-\rho {\left(V_{j}-V_{o}\right)}^{2}S_{j}-\rho (V_{d}{}^{2}+V_{o}{}^{2}+2V_{d}V_{o}\cos\theta )S_{d}\cos\theta ]/S_{o} \end{array}.$$ (8)

According to the Bernoulli differential equation, the stroke pressure in the back chamber of the cylinder can be obtained, as shown in Formula (9).

$$\begin{array}{l} P_{bo}=P_{o}+\frac{1}{2}\rho V_{o}{}^{2}-\frac{1}{2}\rho V_{p}{}^{2}\\ \;=[(\frac{1}{2}\xi_{9}\rho V_{d}{}^{2}+\frac{1}{2}\xi_{10}\rho V_{10}{}^{2}+\frac{1}{2}\xi_{11}\frac{L_{11}}{D_{h11}}V_{11}{}^{2}+P_{b})S_{d}\cos\theta -\rho {\left(V_{j}-V_{o}\right)}^{2}S_{j}\\ \;-\rho (V_{d}{}^{2}+V_{o}{}^{2}+2V_{d}V_{o}\cos\theta )S_{d}\cos\theta ]/S_{o}+\frac{1}{2}\rho V_{o}{}^{2}-\frac{1}{2}\rho V_{p}{}^{2} \end{array}$$ (9)

where *V*_*p*_ represents the velocity of the fluids that flow through the back chamber of the cylinder, which equals to the moving velocity of the piston and hammer.

Depending on the pressure variation of fluids in the jet actuator, the driving force of the hydro-hammer with a jet actuator can be timely observed and analyzed, and the impact energy of the hydro-hammer can be obtained by integrating the driving force and the stroke, which contributes to designing and improving the impact energy of the hydro-hammer.

## 3. Design and numerical simulation

**Fig 3:**
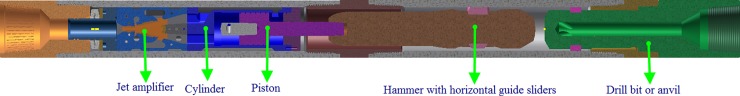
Dimensional structure diagram of the hydro-hammer with a jet actuator.

To improve the penetration rate of directional well drilling in hard rock formations, the designed hydro-hammer with a jet actuator has been attached to a screw motor, which enables the orientation of directional drilling tools. To increase the impact energy and service life of the hydro-hammer, the jet actuator is manufactured with cemented carbide, and the other components of the hydro-hammer are manufactured with 40CrMo. Additionally, the external diameter of the hydro-hammer has been set to 203.2 mm, which is suitable for boring a hole with a diameter of 241–311.5 mm. The moving stroke of piston ranges from 20 mm to 100 mm, and the expected impact energy of this hydro-hammer with a jet actuator reaches up to 400 J. The dimensional structure diagram of the hydro-hammer with a jet actuator is shown in Fig 3. In the present study, the influence of the pumped flow rates and moving stroke on the impact energy of the hydro-hammer has been numerically analyzed.

### 3.1. Numerical model and meshing

The numerical model is established according to the original structure of the hydro-hammer with a jet actuator, and the dimensions of the designed hydro-hammer remain constant. To investigate the influence of input flow rates on the improvement of impact energy, various flow rates are employed in the numerical simulation with computational fluid dynamics (CFD). Similarly, the moving stroke of the piston and hammer is also considered in the simulation. The pressure variation of the drilling fluids flowing through the cylinder lid, which has no added influence on the accuracy of the numerical simulation results, can be ignored because the drilling fluids flowing through the annular space of the drill bit are connected to the Earth’s surface.

**Fig 4:**
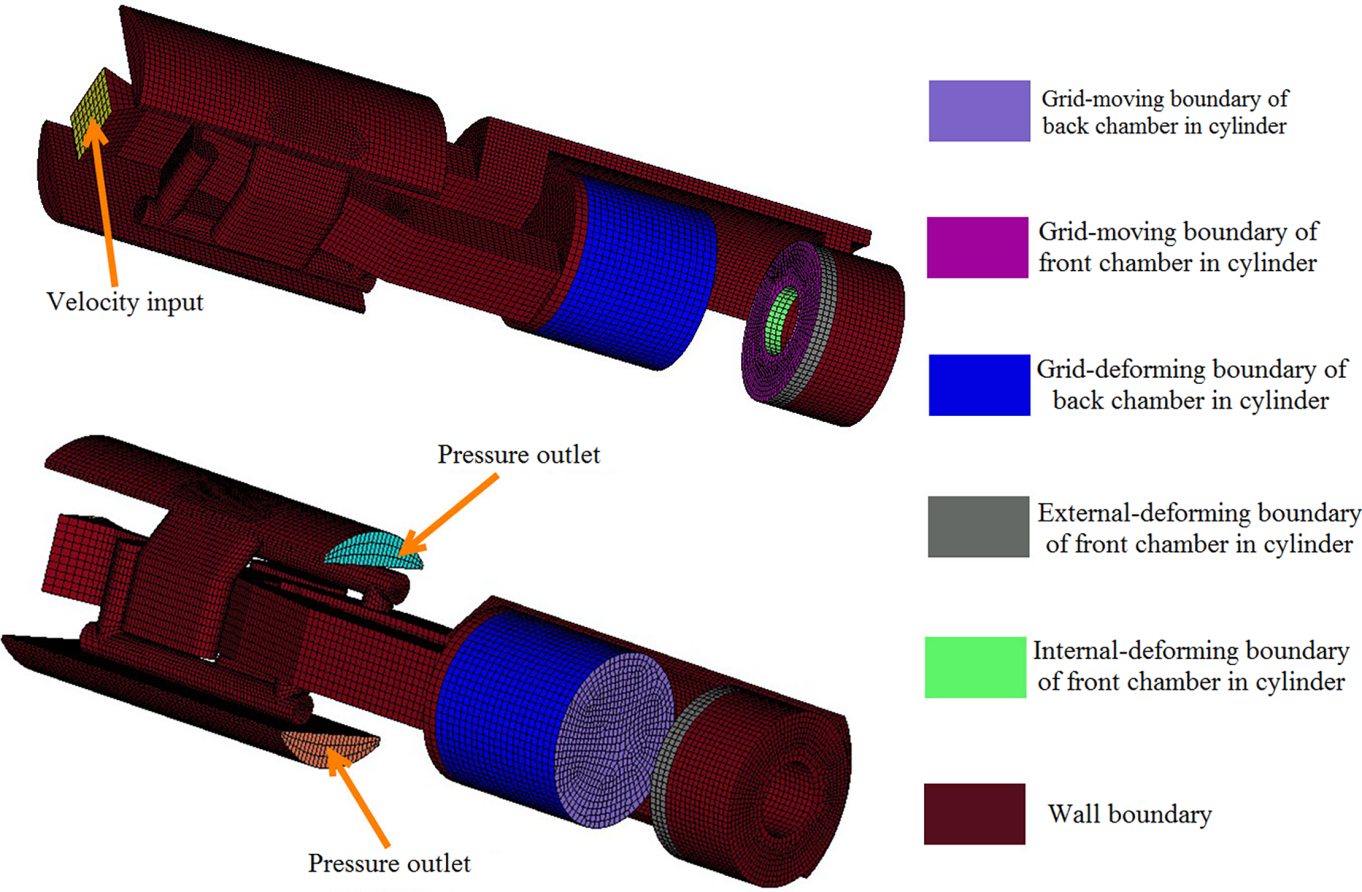
The meshing model of the hydro-hammer with a jet actuator.

The computational fluidic domains in the hydro-hammer with a jet actuator have been meshed by Altair Hypermesh^®^, as shown in Fig 4. A total of 89032 grids have been meshed, including 88429 hexahedral grids and other pentahedron grids. The mesh model of the hydro-hammer is mainly composed of the fluids in the central passage of the jet actuator, in the back chamber of the cylinder, in the output and discharge channels, in the front chamber of the cylinder, flowing through the cylinder lid, and in the annular space of the hydro-hammer. To reduce the difficulty in meshing and restoring, the anomalous flow domain inside the hydro-hammer has been simplified as the inerratic flow domain, which facilitates the improvement of the computing efficiency in the CFD numerical simulation.

In addition, grid sensitivity analysis has been carried out for all grids for one of the computational domains. As shown in Table 1, the maximum difference between the simulation results is less than 15%. Thus, the simulation results are independent of grid size. To calculate the speed and accuracy of the solution, all computational domains have been accomplished by using medium grids.

**Table 1:** The details of the grid sensitivity analysis for a computational domain.

Grids	Number of cells	Average velocity of the jet in flushing nozzle (m/s)	Average moving velocity of the hydro-hammer(m/s)
Coarse grids	47,438	92.81	2.29
Medium grids	89,032	94.29	2.39
Fine grids	113,562	94.61	2.58
Difference		1.90%	11.24%

### 3.2. Boundary conditions

The meshed grids of the computational domain in a hydro-hammer mainly include the volume and surface grids. The flow domain is defined as volume grids, and the initial boundary condition, wall boundary, and deforming domain are defined as surface grids. In the horizontal motion of the piston and hammer, the deforming wall meshes under the piston rod are constantly moving. Hence, the number of wall boundaries in the computational flow domain is increasing. Therefore, numerical simulation methods with dynamic meshes have been employed in the simulation of a hydro-hammer with a jet actuator.

The computation of the numerical simulation has two stages: steady calculation and unsteady calculation. In the steady and unsteady calculation, the inlet boundary conditions of the meshed model are defined as the velocity inlet boundary condition, whereas the pressure–outlet boundary condition is applied to the definition of the outlet boundary. Moreover, the initial value of the pressure-out boundary is assigned as a standard atmospheric pressure. To enhance the wall-attachment stability of the jet in the nozzle, the pressure of the lateral space connected to the back chamber of the cylinder is two times the atmospheric pressure. The initial boundary parameters of the hydro-hammer with a jet actuator is presented in Table 2.

**Table 2:** Initial boundary parameters of CFD meshes in hydro-hammer with a jet actuator.

Flow rate (m^3^/min)		1.5	1.7	1.9		2.1	2.3
Inlet	Area (mm^2^)	2250					
	Velocity (m/s)	11.1	12.6	14.1		15.5	17.1
	Hydraulic diameter	47.36mm					
	Reynold number	401908	456220	510532		561223	619155
	Turbulence intensity	3.19%	3.14%	3.09%		3.06%	3.02%
Outlet	Area (mm^2^)	3385					
	Velocity (m/s)	7.38	8.36	9.35		10.33	11.31
	Hydraulic diameter	7.3mm					
	Reynolds number	41188	46657	52182		57652	63121
	Turbulence intensity	4.24%	4.17%	4.11%		4.06%	4.01%

### 3.3. User-defined function and solver settings

In the CFD numerical simulation with a hydro-hammer, the user-defined functions (UDF) are compiled to describe the instantaneous position of the moving rigid surface. To update the dynamic mesh models, the pressure, comprehensive force, accelerated speed, velocity, and displacement of the piston in the hydro-hammer are compiled into the UDF, and the governing equations are called by the Fluent solver real time.

To observe the distribution of pressure and flow field in the hydro-hammer with a jet actuator, the semi-implicit method for pressure-linked equations (SIMPLE) algorithm is applied to the CFD numerical simulation of the hydro-hammer. Given the specific solution of CFD numerical simulation with the first-order upwind, the kinetic energy and dissipation rate of the turbulent flow in the hydro-hammer can be obtained by the discretization method, which is applicable to most problems on turbulent flow.

However, the solution accuracy is determined by the density of grid cells. Thus, the motion period of the piston and hammer, the jet attachment stability of the jet amplifier, and the computational time must be considered in the time-step settings. According to the previous simulation and calculation results of hydraulic hammers, the alternation of the jet attached to any side of the jet amplifier can be finished in 1 ms during a single impact period of the hydro-hammer. Thus, the desired time-step is set to 1×10^−4^ *s*, that is, approximately 10 to 15 positions of the jet and 300 to 1200 positions of the moving piston will be captured in one calculation step.

### 3.4. CFD simulation results

**Fig 5:**
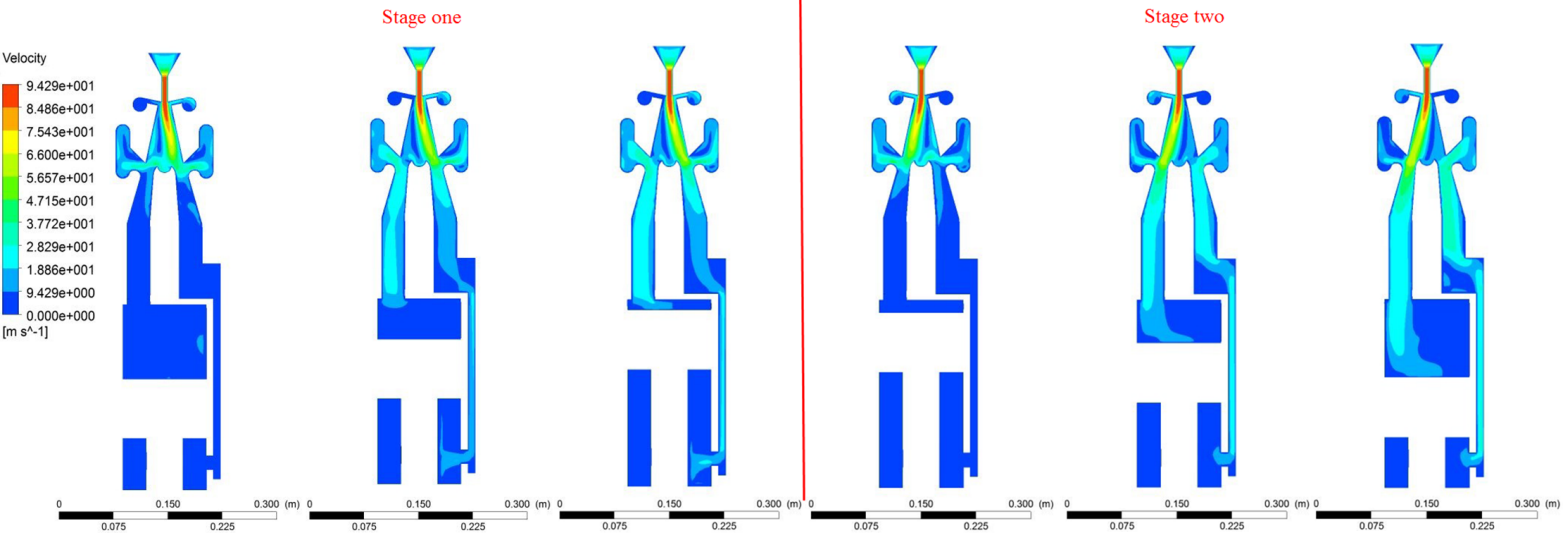
Velocity contour of the accomplished alternation of attached jet in the actuator.

The influence of the pumped flow rate and impact stroke on the impact energy of the hydro-hammer with a jet actuator has been numerically studied, and the impact velocity of each single motion of the piston can also be acquired. According to the numerical simulation results, the designed hydro-hammer with a jet actuator has a stable working performance, and the alternation of the attached jet can be easily accomplished, as shown in Fig 5. Thus, the hydro-hammer with a jet actuator can regularly work with a constant period, and the maximum velocity of the jet actuator is nearly 95 m/s, which is essential for the stable attachment of the jet. Finally, the influence of the variation of the impact velocity and pressure loss on various flow rates and impact strokes of the hydro-hammer has been obtained. In addition, the working frequency of the hydro-hammer with a jet actuator is also analyzed and discussed.

**Fig 6:**
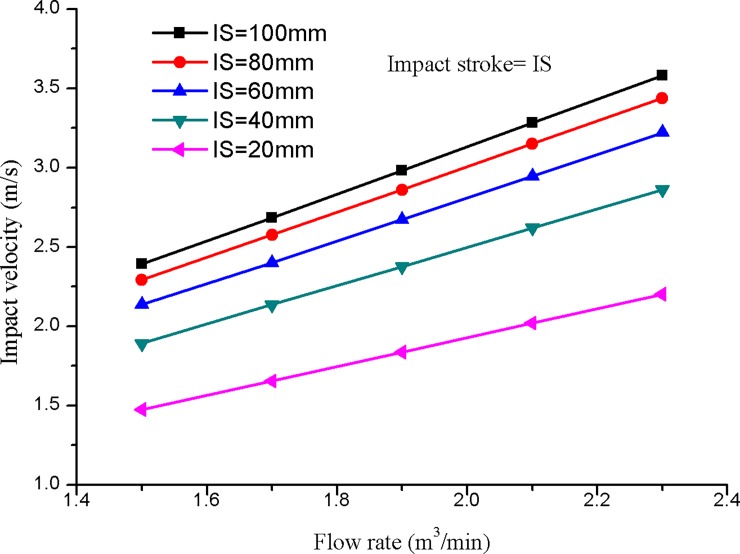
Variation of the impact velocity on various input flow rates.

As shown in Fig 6, the impact velocity of the hammer is numerically calculated with diverse impact strokes and input flow rates. The impact stroke of the hydro-hammer with a jet actuator is 100 mm, and the simulated impact velocity of the hammer varies with the increase of the input flow rate. Thus, the impact velocity of the hammer varies linearly with the increase of the input flow rates. Impact velocity is positively related to the impact stroke of the hydro-hammer with a jet actuator. The maximum impact velocity of the hammer is approximately 3.2 m/s, whereas that of the impact stroke is 100 mm, which is actuated with a flow rate of 2.4 m^3^/min.

According to the numerical simulation results, although the impact stroke of the hammer is 20 mm, the variation slope of the impact velocity is slightly smaller than that of the other impact strokes. In general, the impact stroke has a primary influence on the improvement of the hydro-hammer’s impact energy.

**Fig 7:**
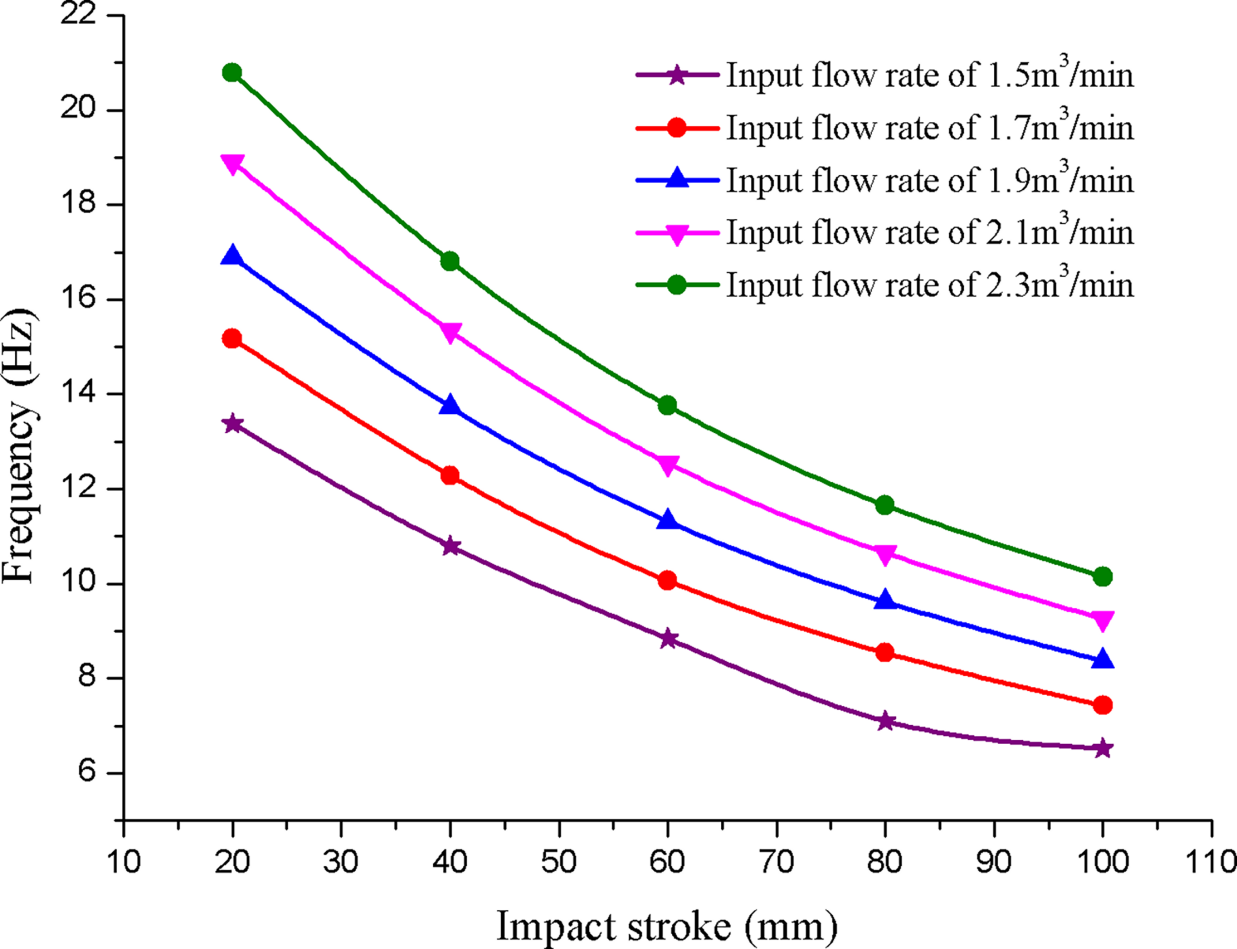
Variation curves of the motion frequency of hydro-hammer with various impact strokes.

The motion frequency of the hydro-hammer is still affected by the impact stroke and input flow rate. As shown in Fig 7, the motion frequency of the hammer reduces with the increase of impact stroke, whereas the motion frequency increases with the input flow rate when the impact stroke of the hammer is constant. The numerically simulated motion frequency of the hydro-hammer with a jet actuator is varies exponentially with the impact stroke, and the maximum and minimum motion frequencies are 21 Hz and 7 Hz, respectively.

**Fig 8:**
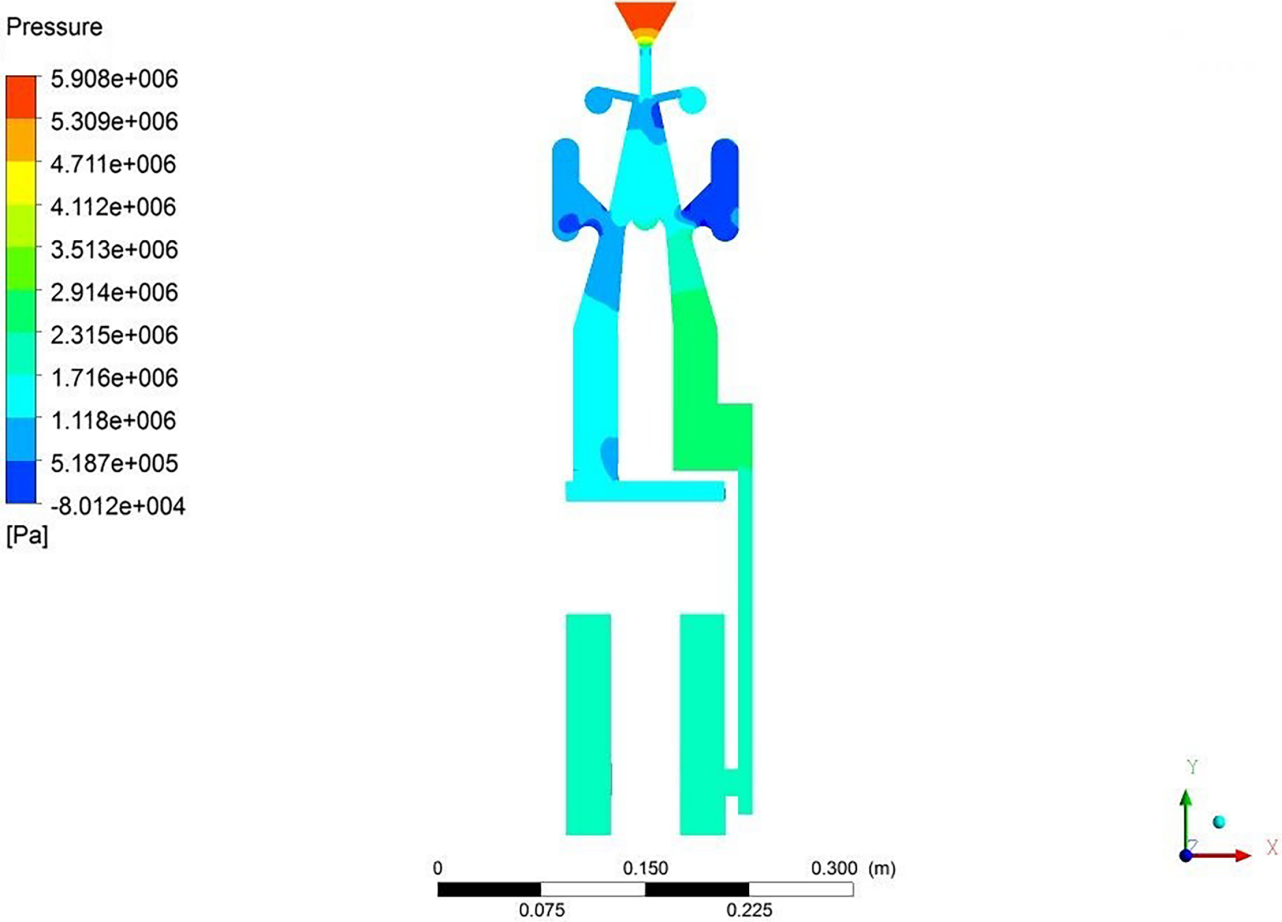
Pressure distribution of the jet actuator with the impact stroke of 100 mm and input flow rate of 2.3 m^3^/min.

In the pressure distribution of the hydro-hammer with a jet actuator during horizontal directional well drilling, the pressure loss of the jet actuator is critical for the improvement of the impact energy. Therefore, the pressure distribution of the jet actuator should be continuously observed. As shown in Fig 8, the maximum pressure of the nozzle in the jet actuator is approximately 5.9 MPa, whereas the impact stroke of the hammer is 100 mm, with an input flow rate of 2.3 m^3^/min.

In addition, the outlet pressure of the jet actuator is nearly 2.3 MPa, and thus the pressure loss of the jet actuator is 3.6 MPa, which means that more energy is required to conquer the hydraulic resistance of the hydraulic hammer. On the basis of the CFD numerical simulation results, the pressure of the nozzle in the jet actuator decreases with the impact stroke and input flow rate.

## 4. Experiments on the hydro-hammer with a jet actuator

To investigate the influence scope of the hydro-hammer on impact energy, laboratory experiments have been conducted with different input flow rates and impact strokes of the hammer. To improve the measurement accuracy of the impact energy of the hydro-hammer, a new measuring system with eddy current type transducers has been set up, and the impact energy of the hydro-hammer with a jet actuator has been obtained.

**Fig 9:**
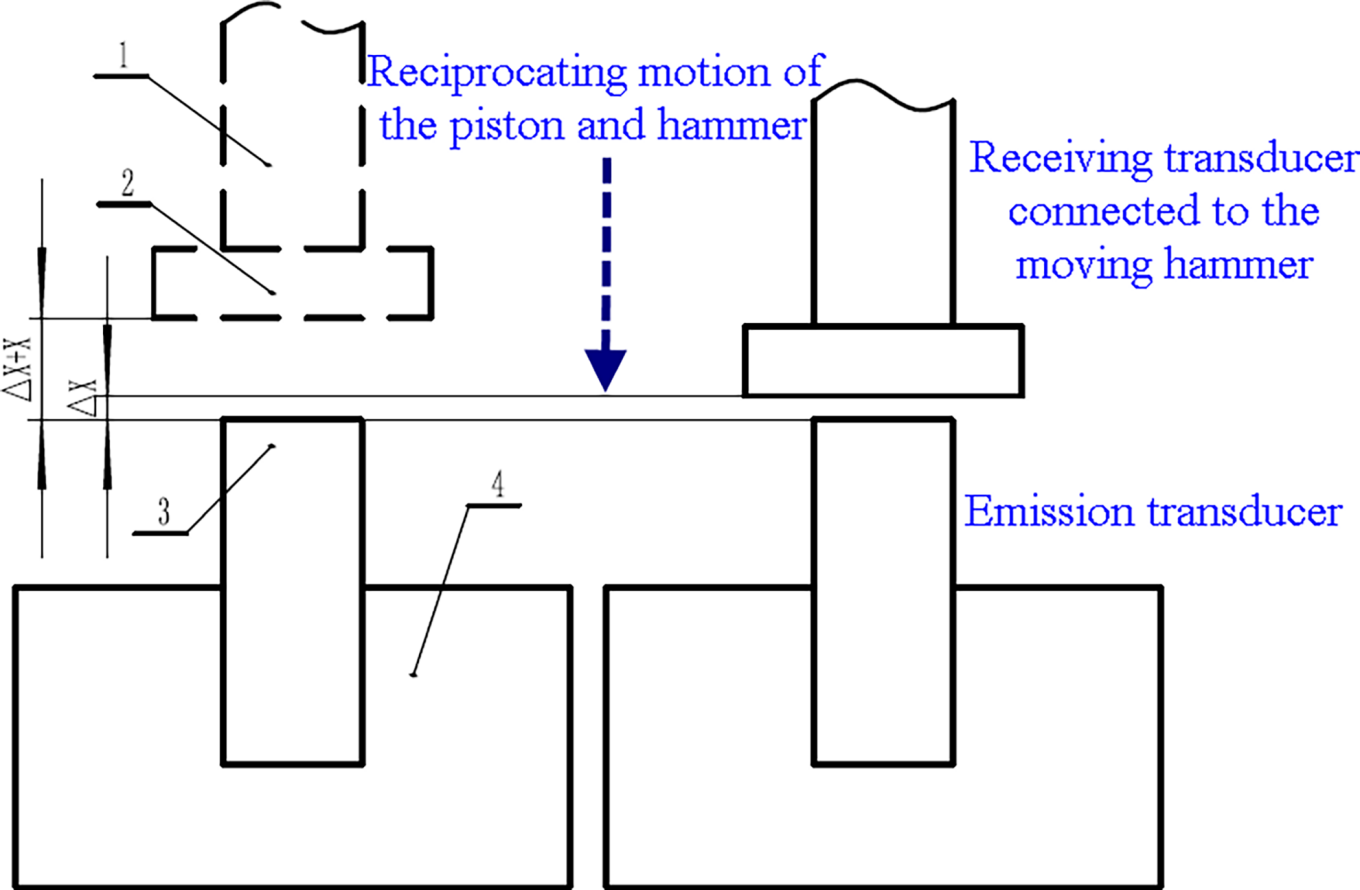
Schematic diagram of the new measuring system with eddy current type transducers.

The new measuring system with eddy current transducers has more precise and frequent impact energy measurement than the conventional measurement system of impact energy. The working mechanism of the new measurement system is based on Faraday's law of electromagnetic induction, as shown in Fig 9. The receiving transducer moves with the piston and hammer, and the induced eddy current is generated, thereby producing an alternating magnetic field with an opposite phase. Therefore, the emission transducer simultaneously outputs singles of electric current or voltage. The output electric current or voltage varies with the distance between the receiving and emitting transducer. Once the receiving transducer moves to distance Δ*x* + *x*, the transient time (*t*_1_) is recorded, and the other transient time (*t*_2_) is obtained while the receiving transducer moves to the end of the hammer’s stroke, which has a distance of Δ*x*. Hence, the impact velocity of the piston or hammer can be calculated by the following formula: *x*/(*t*_2_−*t*_1_).

**Fig 10:**
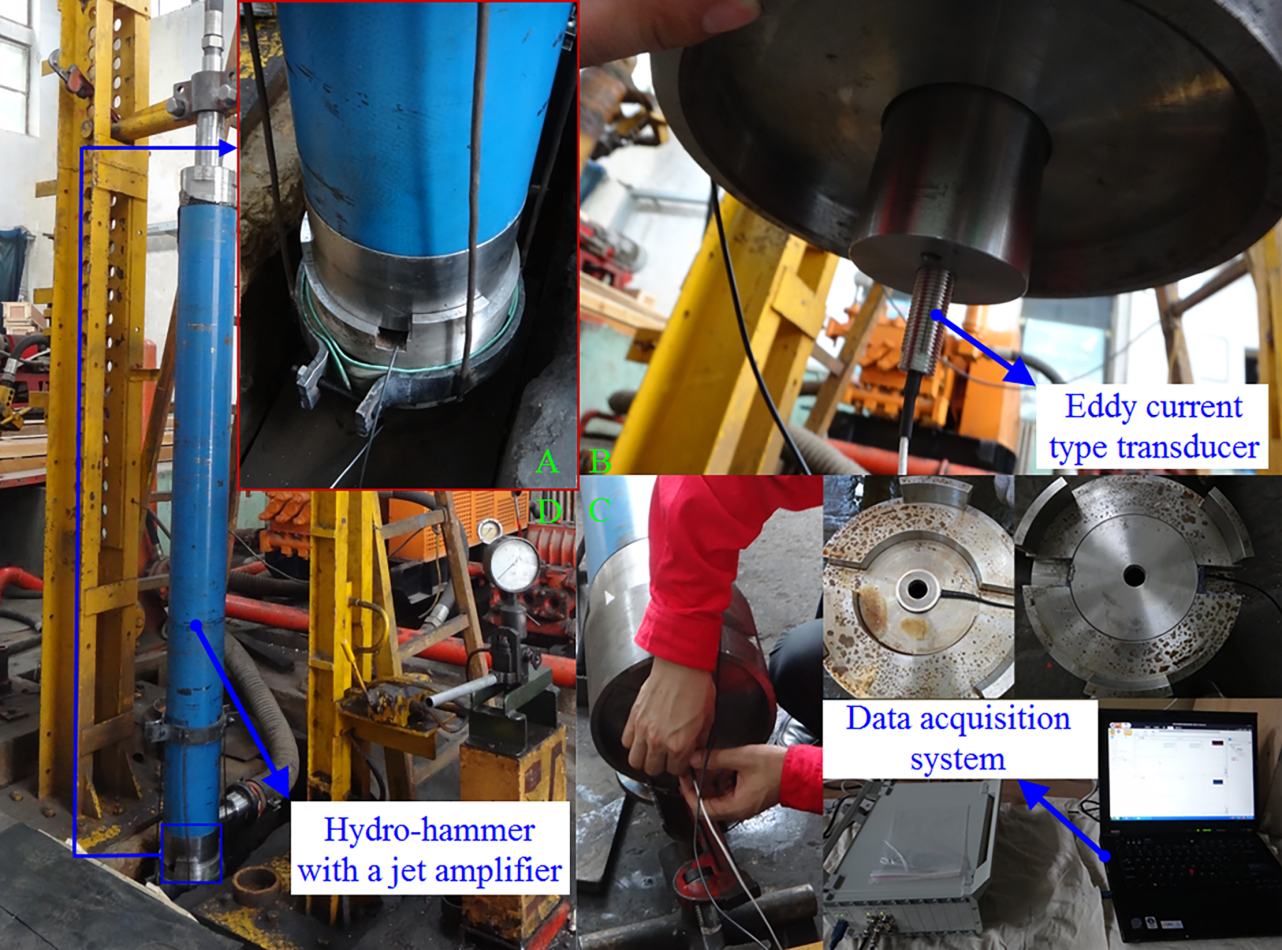
Experiments apparatus of the measurement on impact velocity of hydro-hammer with a jet actuator.

As shown in Fig 10, the experimental apparatus of the measuring system mainly consists of the prototype of a hydro-hammer, the data acquisition system, eddy current transducers, and the accessory system of drilling, such as the high-pressure pump and tube. Finally, the impact velocity of the piston in the hydro-hammer has been measured with diverse flow rates and impact strokes. A total of 25 groups of impact velocities of the hydro-hammer have been measured with different working parameters, and the measurement data have been recorded and analyzed.

The impact stroke of the hammer is 100 mm, the impact velocity varies with the increase of the pumped flow rates, and the working position of the hydro-hammer is excellent. The measured performance parameters of the hydro-hammer with a jet actuator are listed in Table 3. The average motion velocity of the hammer is 2.6 m/s, and the maximum impact velocity of the hammer reaches up to 3.19 m/s, which is faster than the former valved hydraulic hammers. The motion velocity of the hammer slightly increases with the increase of pumped flow rate. In addition, the maximum impact energy of the hydro-hammer with a jet actuator is 407 J, whereas the input flow rate is 2.3 m^3^/min.

**Table 3:** The measured performance parameters of the hydro-hammer with a jet actuator while the impact stroke of the hammer is 100mm.

Flow rates (m^3^/min)	Pressure loss (MPa)	Average velocity (m/s)	Impact energy (J)	Motion frequency (Hz)
1.5	5	2.13	181.476	6
1.7	5	2.32	215.296	7
1.9	5.5	2.54	258.064	7
2.1	6	2.83	320.356	7
2.3	6.5	3.19	407.044	8

**Fig 11:**
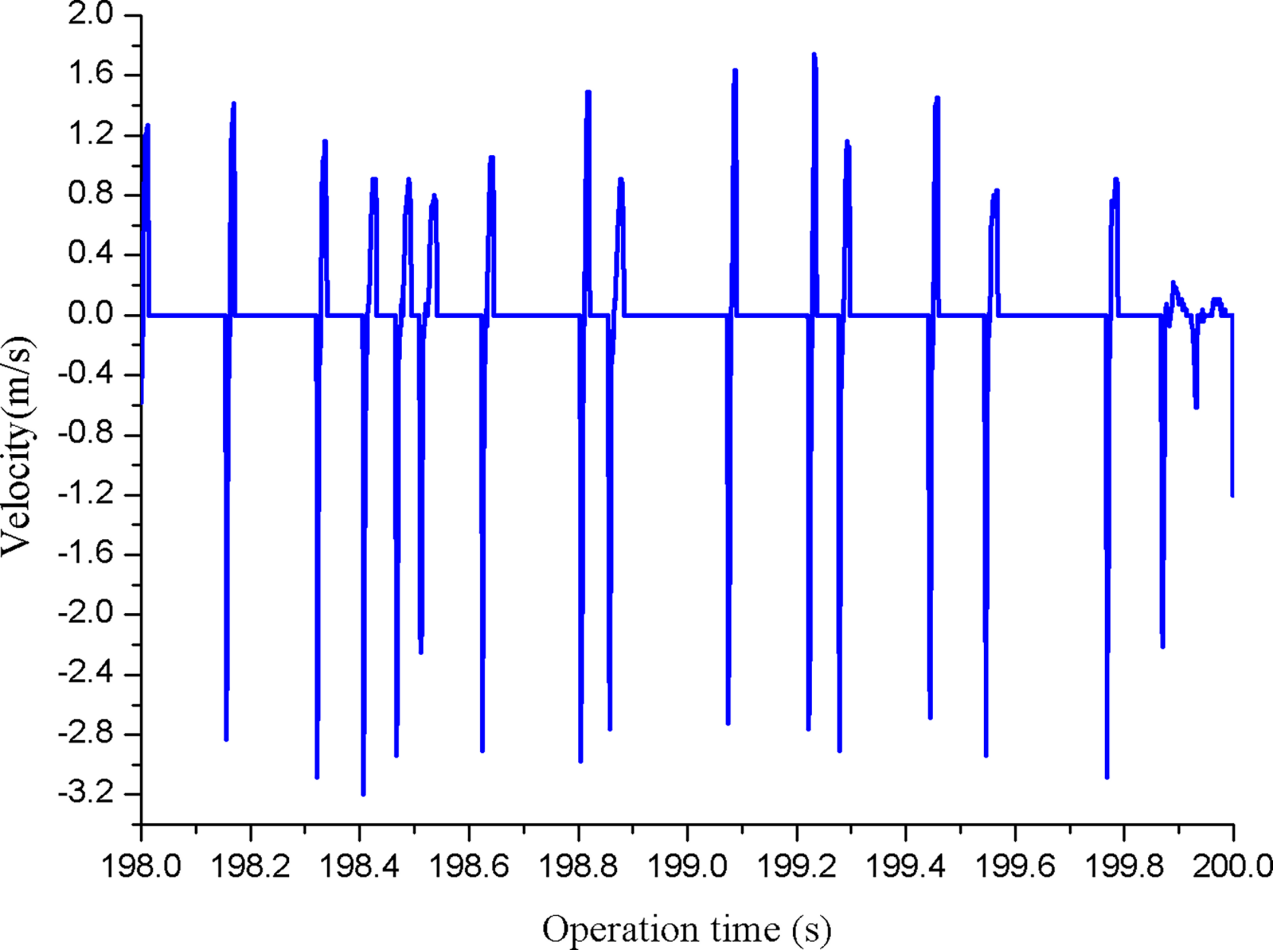
The variation of the motion velocity of hydro-hammer on operation time while the impact stroke is 100 mm.

The impact stroke of the hydro-hammer with a jet actuator is 100 mm, and the variation of the motion velocity on operation time of the hammer is shown in Fig 11. Thus, the impact velocity of the hammer slightly fluctuates with the increase in operation time. The motion frequency of the hammer is approximately 7 Hz on the basis of the acquired velocity variation curve, and the maximum motion frequency of the hydro-hammer is 8 Hz, and the input flow rate is 2.3 m^3^/min. The impact energy of the hydro-hammer with a jet actuator is approximately identical, because each impact duration is balanced, and thus the hydro-hammer exhibits good performance in the experiments.

**Fig 12:**
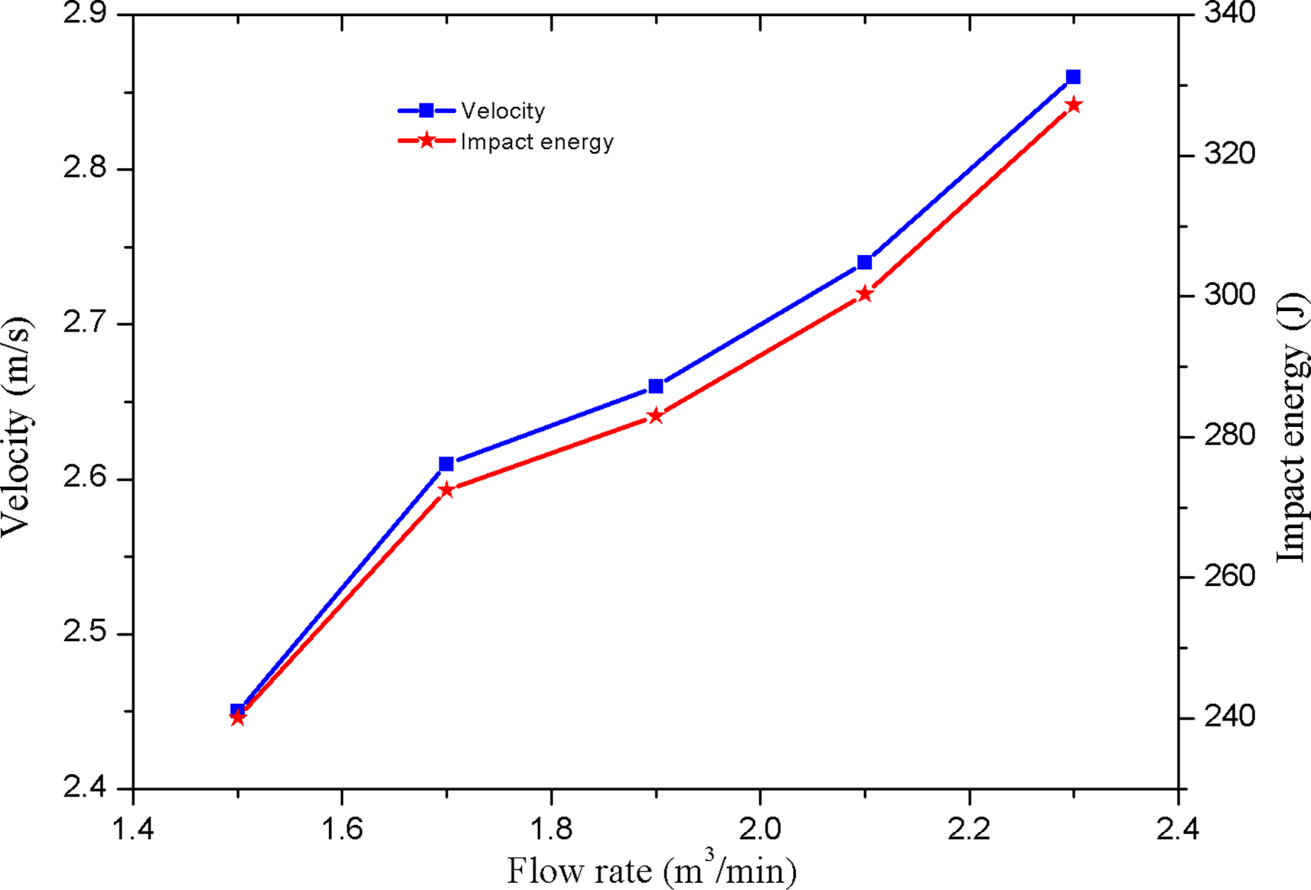
Variation on velocity and impact energy of the hammer while the impact stroke is 80 mm.

As shown in Fig 12, the variation of the velocity and impact energy of the hydro-hammer with a jet actuator is influenced by the input flow rate. The impact stroke is 80 mm, the average motion velocity of the hammer is 2.66 m/s, and the maximum impact velocity of the hammer reaches to 2.93 m/s. The impact velocity and energy of the hammer increase with the pumped flow rate. Compared to the velocity variation of the hydro-hammer with a 100-mm impact stroke, the deviation between the average motion velocity and the maximum velocity of the hammer is minor, which means that the working performance of the hydro-hammer with a jet actuator is superior.

The impact stroke of the hammer is 60 mm, and the variation of the motion velocity and impact energy of the hammer is listed in Table 4. The average motion velocity of the hammer is 2.38 m/s, and the maximum impact velocity of the hammer reaches to 2.69 m/s. Thus, the average motion velocity of the hammer decreases with the impact stroke of the hammer. With the increase in pumped flow rate, the motion velocity of the hammer slightly increases, but the impact stroke is identical. The impact stroke is 60 mm, the maximum impact energy of the hydro-hammer with a jet actuator is 287 J, and the input flow rate is 2.3 m^3^/min. According to the obtained experiments results, the motion frequency of the hydro-hammer decreases with the impact stroke. In addition, the motion frequency deviation of the hydro-hammer is barely influenced by the input flow rate. The pumped flow rate changes from 1.5 m^3^/min to 2.3 m^3^/min, and the motion frequency of the hammer just ranges from 6 Hz to 8 Hz. The stable motion of the hydro-hammer with a jet actuator is advantageous for the performance improvement of the hydro-hammer.

**Table 4:** The variation of motion velocity and impact energy of the hammer while the impact stroke is 60mm.

Flow rate (m^3^/min)	Pressure loss (MPa)	Average velocity (m/s)	Impact energy (J)	Motion frequency (Hz)
1.5	4	2.13	181.476	8
1.7	5	2.28	207.936	6
1.9	5	2.35	220.9	7
2.1	5.5	2.49	248.004	8
2.3	5.5	2.68	287.296	8

**Fig 13:**
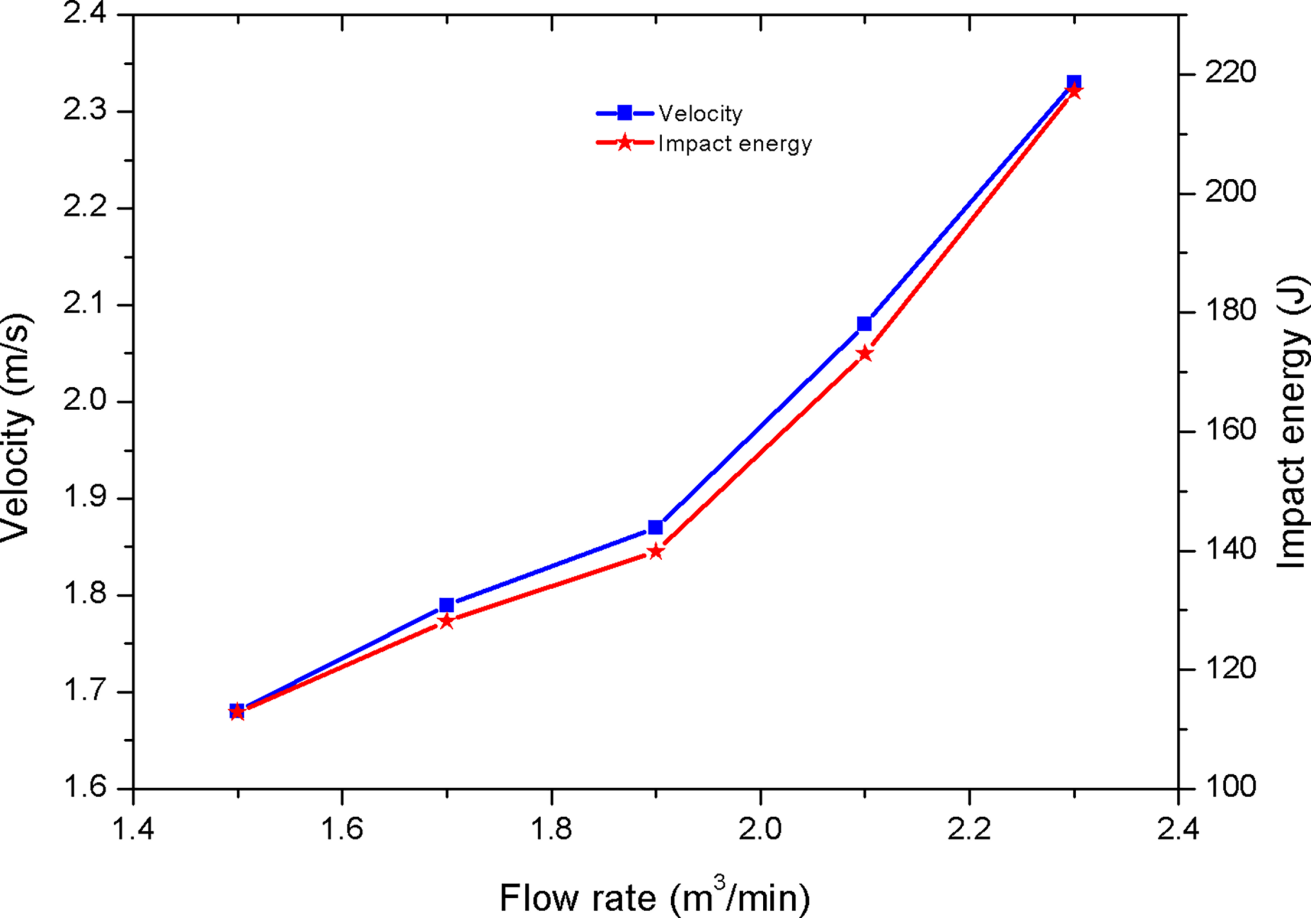
Variation on velocity and impact energy of the hammer while the impact stroke is 40 mm.

As shown in Fig 13, the maximum impact velocity of the hammer is only 2.36 m/s, and the impact stroke is 40 mm and the pumped flow rate is 2.3m^3^/min. The average velocity and impact energy of the hammer increase with the increase of the pumped flow rate. Therefore, the working stability of the hydro-hammer may be improved by increasing the pumped flow rate, which represents the attachment and conversion performance of the jet in the bi-stable jet actuator has been optimized. In addition, the average velocity and impact energy of the hammer are dramatically varied, while the pumped flow rate is more than 1.9 m^3^/min. According to the variation curve of the velocity and impact energy of the hammer, the pumped flow rate is proposed to be 1.9 m^3^/min to improve the impact energy of the hydro-hammer with a jet actuator.

**Fig 14:**
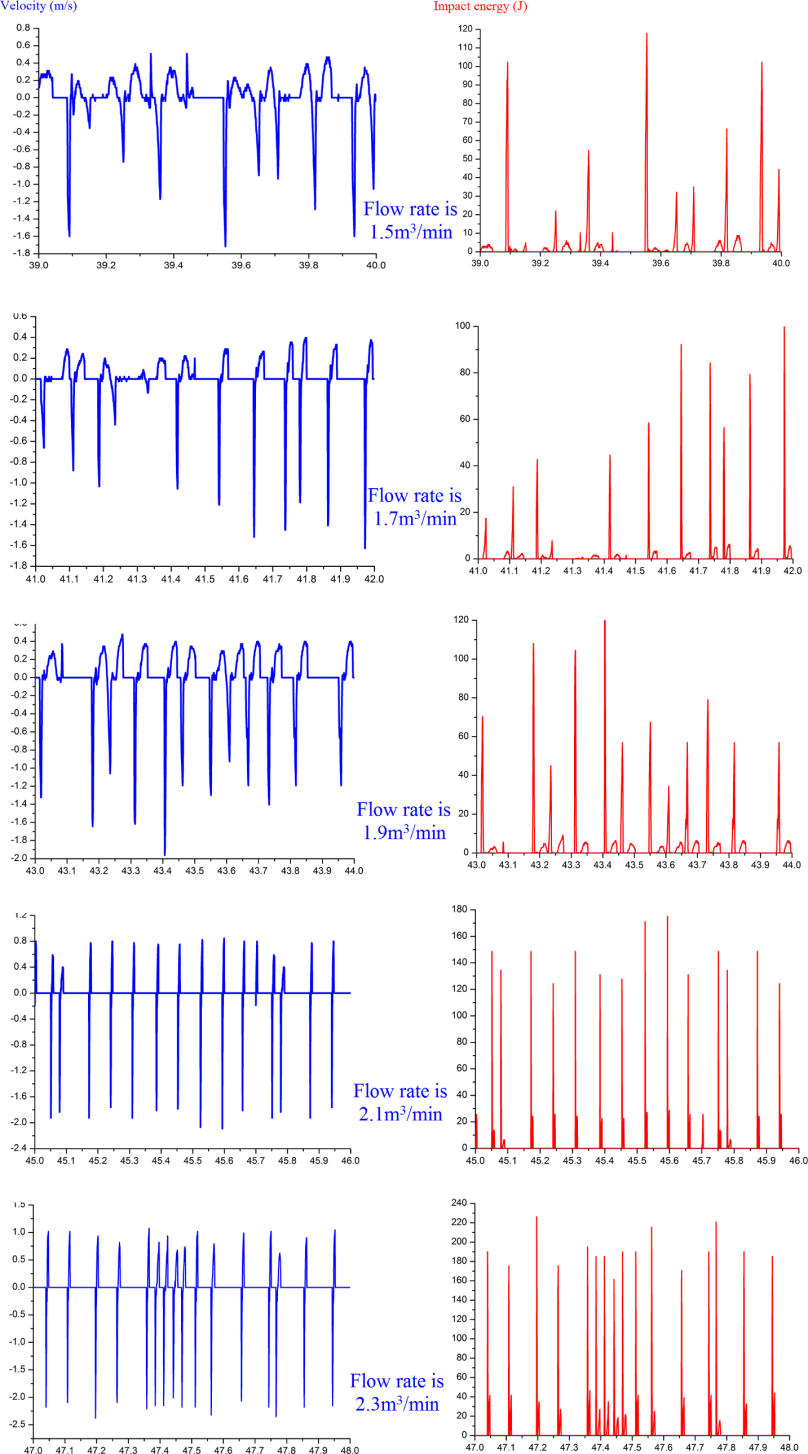
The measured variation of real-time velocity and impact energy while the impact stroke of hammer is 40 mm.

During the working process of the hydro-hammer with a jet actuator, the measured variation of real-time velocity and impact energy is presented in Fig 14. According to the measured results, the deviation of impact velocity and energy is minimal, and the pumped flow rate of the hydro-hammer is 2.1 m^3^/min. Each wave crest denotes an entire impact process of the hydro-hammer with a jet actuator, and thus the working performance can be graphically demonstrated by the measured impact velocity and energy. Therefore, the motion frequency of the hydro-hammer ranges from 10 Hz to 16 Hz, while the impact stroke of the hammer is 40 mm.

The impact stroke of the hydro-hammer with a jet actuator is 20 mm, and the variation of the average motion velocity and impact energy on pumped flow rate is shown in Table 5. The motion velocity and impact energy of the hammer is sharply reduced compared to the working performance of the hydro-hammer with an impact stroke of 40 mm. The average velocity and impact energy slightly increase with the pumped flow rate because the pumped flow rate is less than the nominal flow rate of the hammer. The maximum impact energy of the hammer is 131 J, and the pumped flow rate is 2.3 m^3^/min, and the maximum motion velocity is 1.81 m/s. According to the measured motion velocity and impact energy of the hydro-hammer with a jet actuator, the deviation of velocity and energy is tremendous, which indicates the unstable working performance of the hammer.

**Table 5:** Performance parameters of the hydro-hammer while the impact stroke is 20 mm.

Flow rate (m^3^/min)	Pressure loss (MPa)	Average velocity (m/s)	Impact energy (J)	Motion frequency (Hz)
1.5	2.2	1.18	55.696	12
1.7	2.6	1.31	68.644	14
1.9	4	1.52	92.416	16
2.1	4	1.64	107.584	18
2.3	4.8	1.81	131.044	20

The motion frequency of the hydro-hammer with a jet actuator ranges from 12 Hz to 20 Hz because the impact stroke of the hammer is 20 mm. Therefore, the motion frequency of the hydro-hammer is mainly influenced by the impact stroke. However, the working status of the designed hydro-hammer with a jet actuator is deficient when the impact stroke is 20 mm, but the hydro-hammer is still launched to work with the pumped flow rate of 2.3 m^3^/min.

**Fig 15:**
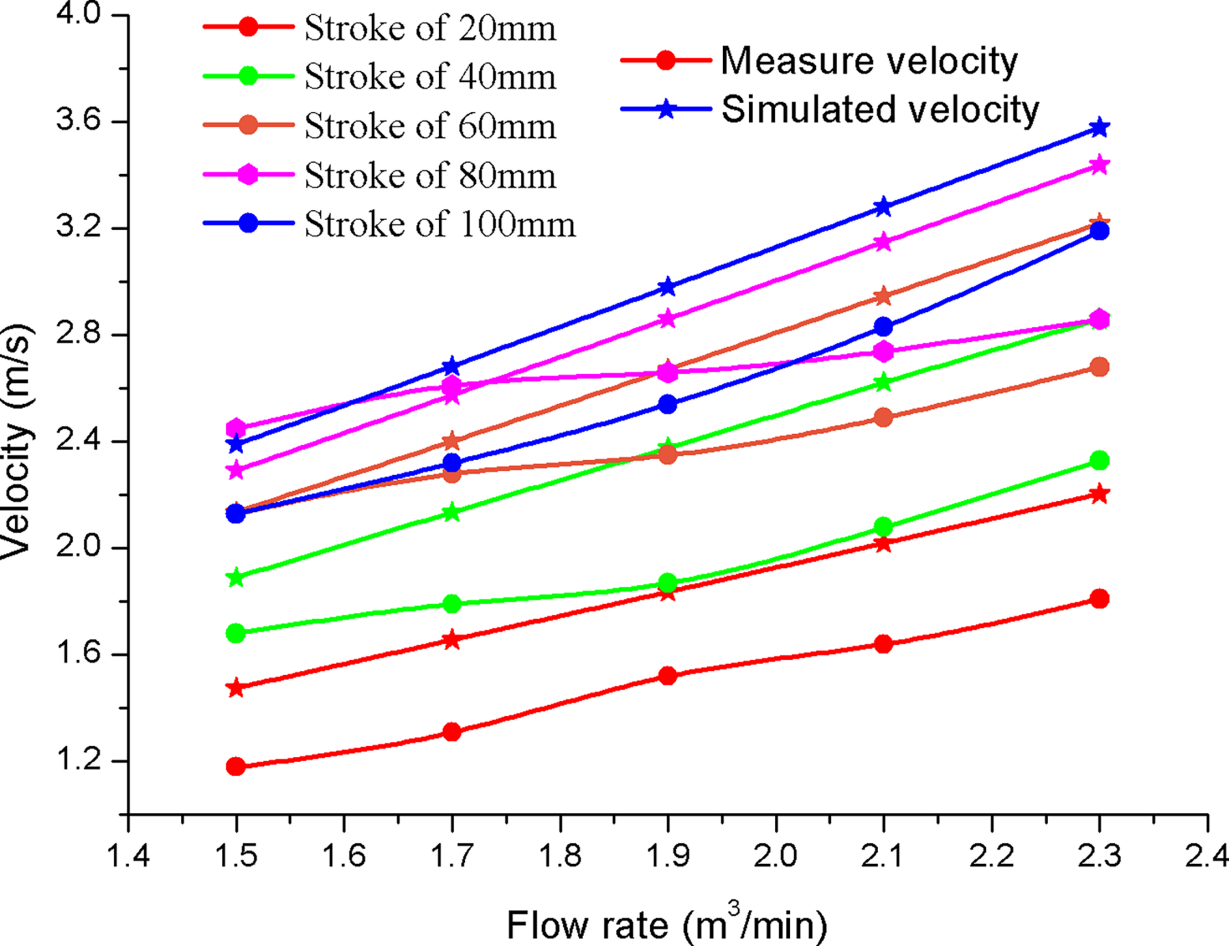
Comparison of motion velocity of the hydro-hammer between measured value and numerical simulation results.

Compared to the numerical simulation results of motion velocity, the measured performance parameters of the hydro-hammer are relatively minor. As shown in Fig 15, experiment and numerical simulation results have shown that the impact velocity of the hammer increases with the pumped flow rate, and the velocity deviation between the simulation results and the measured value ranges from 0.2–0.7 m/s. The measured velocity and impact energy are smaller than the numerical simulation results due to the pressure consumption and flow rate pulsation in the hydro-hammer with a jet actuator.

**Fig 16:**
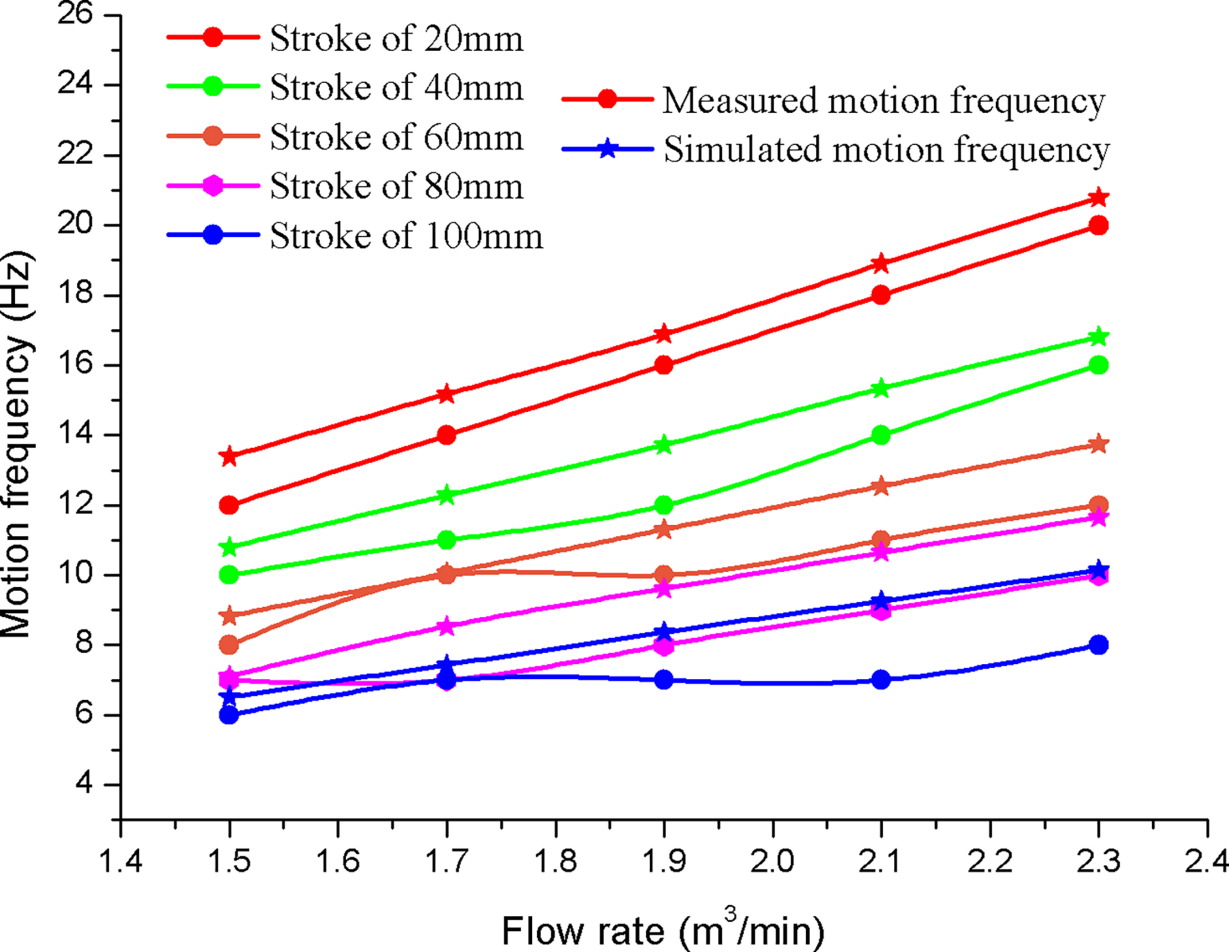
Comparison of motion frequency of the hydro-hammer between measured value and numerical simulation results.

As shown in Fig 16, the maximum deviation of the motion frequency between simulation results and measured value is 1–2 Hz, and the simulated motion frequency of the designed hydro-hammer is greater than the measured value due to the different boundary conditions between the numerical simulation and the laboratory experiments. Therefore, the pumped flow rate and the impact stroke of the hammer have influence on the motion frequency of the hydro-hammer according to the numerical simulation and laboratory experimental results. In addition, the simulated and measured motion frequency of the hammer will increase with the pumped flow rate. The pumped flow rate of the hydro-hammer has a greater influence on the variation of motion frequency.

## 5. Field tests and discussions

To investigate the working performance of the hydro-hammer with a jet actuator, field tests using a hydro-hammer have been conducted in the Dragon River Horizontal Directional Well Drilling Program, which is situated in Yunnan Province, China (EL98° 25′; NL24° 07′). The performance parameters of the hydro-hammer have been comprehensively evaluated according to the simulation and experiments results. Finally, the performance parameters of the hydro-hammer with a jet actuator have been obtained, including the pressure variation and the penetration rate of the hydro-hammer.

**Fig 17:**
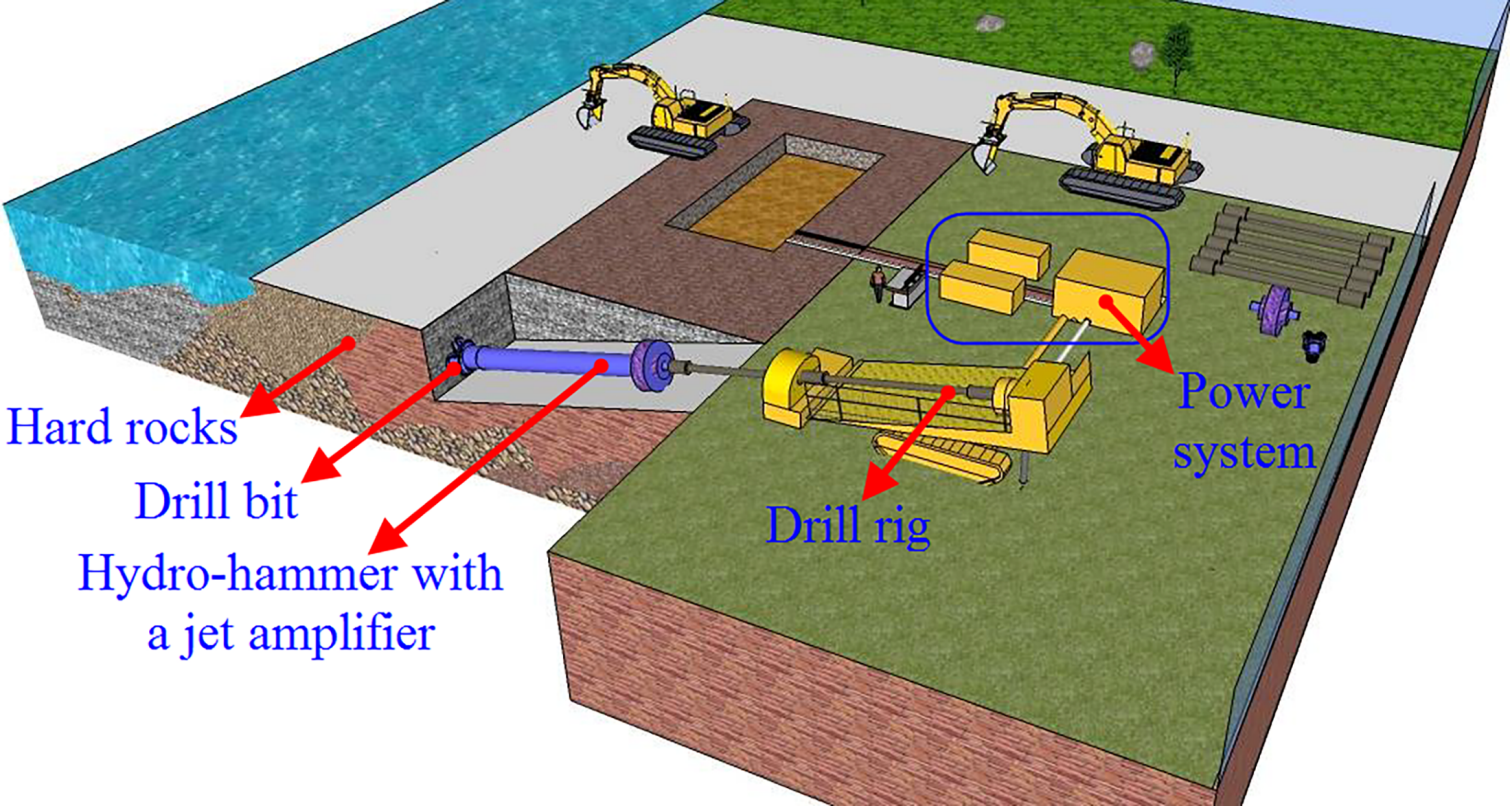
Schematic diagram of the field test configuration while in horizontal directional well drilling with hydro-hammer.

**Fig 18:**
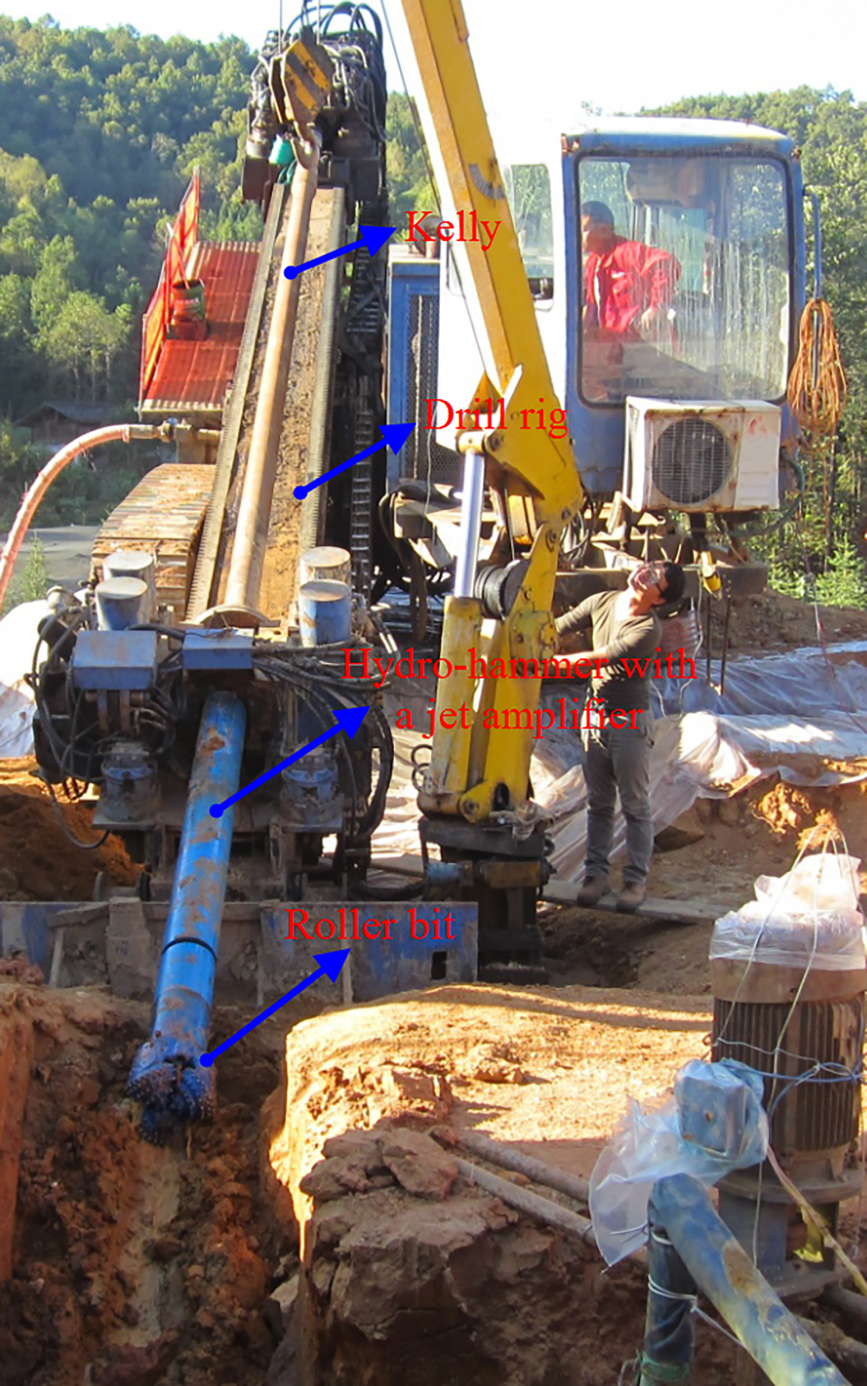
Layout of construction site in horizontal directional well drilling.

The schematic of the field test apparatus configuration with a hydro-hammer is shown in Fig 17. The power system, drill rig, drill bit, hydro-hammer with a jet actuator, and other accessory equipment are included in the field tests. In the targeted formations in horizontal directional well drilling, the drilled granite is weathered to some extent, and the comprehensive strength of the hard formation is approximately 120 MPa. The hydro-hammer with a jet actuator has been operated with the screw motor, which contributes to the accomplishment of orientation. In addition, the borehole in the field tests with a hydro-hammer has been bored with a diameter of 219 mm. The layout of the construction site in horizontal directional well drilling is shown in Fig 18.

During the drilling of the hydro-hammer with a jet actuator, the hammer has a superior performance in hard rock drilling. As shown in Table 6, the maximum penetration rate of the hammer reaches up to 30 m/h, and the average penetration rate is 21.42 m/h. According to previous drilling results, the common penetration rate of the conventional drilling technique is only 16.25 m/h, and thus the penetration rate of horizontal directional well drilling in hard rocks has been improved by 30% when drilling with a hydro-hammer with a jet actuator.

**Table 6:** Field test results of directional well drilling with a hydro-hammer.

No.	Depth of borehole /m	Drilling time /min	Penetration rates /m·h^-1^
1	0–2.5	10	15.00
2	2.5–5.0	8	18.75
3	5.0–7.5	8	18.75
4	7.5–10.0	7	21.42
5	10.0–12.5	6	25.00
6	12.5–15.0	6	30.00
7	15.0–17.5	5	30.00
8	17.5–20.0	6	25.00
9	20.0–22.5	7	21.42
10	22.5–25.0	7	21.42
Total	25.0	70	Average 21.42

## 6. Conclusions

To improve the penetration rate and impact energy of the hydraulic hammer with a jet actuator, the performance of the hydro-hammer has been numerically and experimentally analyzed, and the hydraulic hammer has been applied in rapid directional well drilling in hard rock formations. Therefore, the following conclusions are drawn:

1. A new drilling system with percussive and rotary drilling technology has been proposed and applied to rapid horizontal directional well drilling, and the hydro-hammer with a jet actuator has also been theoretically designed, which facilitates the improvement of penetration rates while drilling in hard rock formations.

2. The impulse hydro-turbine model has been established for investigating the pressure and velocity variation of a hydro-hammer, and the performance parameters of hydro-hammers have also been numerically and experimentally analyzed. Furthermore, the maximum motion velocity and impact energy of the hydro-hammer have been improved, which reaches up to 3.19 m/s and 407 J, respectively.

3. Field tests with a hydro-hammer have been conducted in horizontal directional well drilling. The maximum penetration rate is 30 m/h, and the average penetration rate is 21.42 m/h. Thus, the drilling efficiency has been improved by 30% compared to the conventional drilling technique in hard rock formations.

## Supporting information

S1 Original Data The performance parameters of the hydro-hammer. (XLSX)
